# Developing and Testing the Usability of a Novel Child Abuse Clinical Decision Support System: Mixed Methods Study

**DOI:** 10.2196/51058

**Published:** 2024-03-29

**Authors:** Amy Thomas, Andrea Asnes, Kyle Libby, Allen Hsiao, Gunjan Tiyyagura

**Affiliations:** 1 Department of Pediatrics Yale University School of Medicine New Haven, CT United States; 2 3M | M*Modal 3M Health Information Systems 3M Company Maplewood, MN United States

**Keywords:** child abuse, clinical decision support, CDS, pediatrics, child, children, natural language processing, usability, clinical decision support system, physical abuse

## Abstract

**Background:**

Despite the impact of physical abuse on children, it is often underdiagnosed, especially among children evaluated in emergency departments (EDs). Electronic clinical decision support (CDS) can improve the recognition of child physical abuse.

**Objective:**

We aimed to develop and test the usability of a natural language processing–based child abuse CDS system, known as the Child Abuse Clinical Decision Support (CA-CDS), to alert ED clinicians about high-risk injuries suggestive of abuse in infants’ charts.

**Methods:**

Informed by available evidence, a multidisciplinary team, including an expert in user design, developed the CA-CDS prototype that provided evidence-based recommendations for the evaluation and management of suspected child abuse when triggered by documentation of a high-risk injury. Content was customized for medical versus nursing providers and initial versus subsequent exposure to the alert. To assess the usability of and refine the CA-CDS, we interviewed 24 clinicians from 4 EDs about their interactions with the prototype. Interview transcripts were coded and analyzed using conventional content analysis.

**Results:**

Overall, 5 main categories of themes emerged from the study. CA-CDS benefits included providing an extra layer of protection, providing evidence-based recommendations, and alerting the entire clinical ED team. The user-centered, workflow-compatible design included soft-stop alert configuration, editable and automatic documentation, and attention-grabbing formatting. Recommendations for improvement included consolidating content, clearer design elements, and adding a hyperlink with additional resources. Barriers to future implementation included alert fatigue, hesitancy to change, and concerns regarding documentation. Facilitators of future implementation included stakeholder buy-in, provider education, and sharing the test characteristics. On the basis of user feedback, iterative modifications were made to the prototype.

**Conclusions:**

With its user-centered design and evidence-based content, the CA-CDS can aid providers in the real-time recognition and evaluation of infant physical abuse and has the potential to reduce the number of missed cases.

## Introduction

### Background

Child physical abuse is commonly missed by emergency department (ED) providers, leading to escalating injuries and death [1]. More than 30% of children with serious injuries resulting from physical abuse have been previously evaluated for injuries that were not recognized as abusive [2-5]. This is amplified in general EDs, where most children receive emergency care and abuse is more frequently missed than in pediatric EDs [6-8]. Evaluation and reporting of child abuse are also impacted by provider biases [2,9-12]. Children belonging to racial or ethnic minority groups are more often evaluated for abusive head trauma than White or non-Hispanic children, and children with public insurance undergo increased testing and are reported more often to Child Protective Services (CPS) than privately insured children [9,12]. These findings highlight the need for systems that standardize care, improve clinical outcomes, and reduce bias.

Clinical decision support (CDS) integrated into the electronic health record (EHR) can present intelligently filtered, individualized, and timely information to enhance clinical decision-making [13]. Child abuse–specific CDS systems may improve outcomes and reduce bias in the evaluation and reporting of suspected abuse. Experts have shared consensus recommendations regarding developing, disseminating, and sustaining EHR-embedded child abuse CDS systems in the ED [14]. Key recommendations included universal, routine implementation of a child abuse CDS system in general and pediatric EDs for children aged <4 years; use of active alerts that share their reason for triggering; integration of a standardized system for reports to CPS; use of data warehouse reports to evaluate the CDS system’s efficacy; integration of a system that is feasible, sustainable, and easily disseminated; and personalized usability testing to ensure seamless integration of the system [14].

While reviewing the existing child abuse CDS systems [15-32], we found that a common limitation is their inability to be triggered by free text in an EHR encounter. To address this gap, our team previously developed and validated a natural language processing (NLP) algorithm that automatically and methodically examined the free text in the notes of nursing providers, medical providers, and social workers (SWs) to identify high-risk injuries associated with possible abuse in children [33]. The NLP algorithm would provide a positive alert when it identified preselected combinations of written terms associated with fractures, intracranial injuries, abdominal injuries, burns, bruising, or oral injuries. It was targeted to identify high-risk injuries in children aged <1 year (ie, infants) specifically given that infants are more than twice as likely to experience maltreatment and thrice as likely to experience fatality from maltreatment compared to older children of any age group [1]. Developing a novel child abuse CDS system triggered by this validated NLP algorithm may further increase the tool’s potential to reduce the number of missed cases and mitigate bias.

To change providers’ practice using CDS, it is crucial to understand the providers’ needs and priorities before development and implementation. Evaluating a system’s usability involves the assessment of its accommodation of users’ needs, ease of mastery, effects on workflow, and achievement of goals. Conducting evaluations during the design process is also important to identify shortcomings and incorporate user-centered modifications [34,35]. Usability testing can include direct observation, recording of user-system interactions, *think-aloud* sessions where users verbalize their thoughts while interacting with the system, *near-live* sessions where users test the system with simulated patient interactions, live testing, and quantitative measures [34,36]. However, to date, only 1 study has described the usability testing of child abuse CDS in local settings [31].

### Objective

Therefore, in this study, we aimed to develop a novel child abuse CDS system—hereafter referred to as the Child Abuse Clinical Decision Support (CA-CDS)—which is triggered by a validated NLP algorithm and that both alerts ED providers to high-risk injuries in infants and provides evidence-based recommendations for evaluation and management. We also sought to test the usability of the CA-CDS and refine the system based on user feedback.

## Methods

### Study Design

The study consisted of 3 phases informed by the Guideline Implementation with Decision Support (GUIDES) checklist by Van de Velde et al [37], which describes factors relevant to the development of successful guideline-based CDS. The phases included the (1) development of a prototype, (2) mixed methods usability testing, and (3) iterative refinement of the CA-CDS based on stakeholder feedback. Participants were stakeholders from 4 EDs, including 1 (25%) academic pediatric ED (Yale New Haven Children’s Hospital) and 3 (75%) community pediatric and general EDs (Bridgeport Hospital, Lawrence + Memorial Hospital, and Saint Raphael Campus). All campuses use Epic (Epic Systems Corporation) as their EHR and can use the M*Modal Fluency Direct speech recognition technology and Natural Language Understanding platform (3M Company), which hosts the NLP algorithm and presents the CA-CDS via built-in computer-assisted physician documentation functionality.

### Development of the Prototype

**Figure 1:**
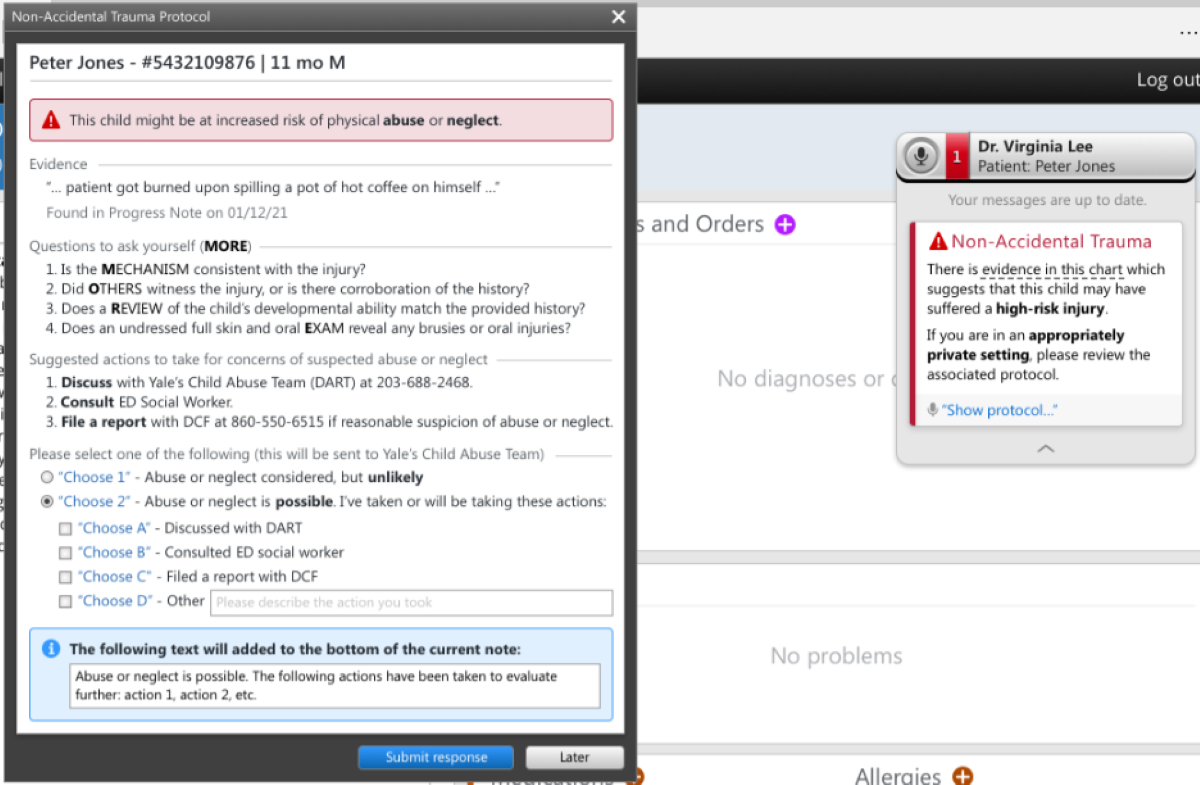
Initial first-provider version of the medical provider–specific Child Abuse Clinical Decision Support (CA-CDS) prototype within a model electronic health record. DART: Detection, Assessment, Referral, and Treatment; DCF: Department of Children and Families; ED: emergency department; M: male; MORE: Mechanism, Others present, Review of development, and Examination details.

**Figure 2:**
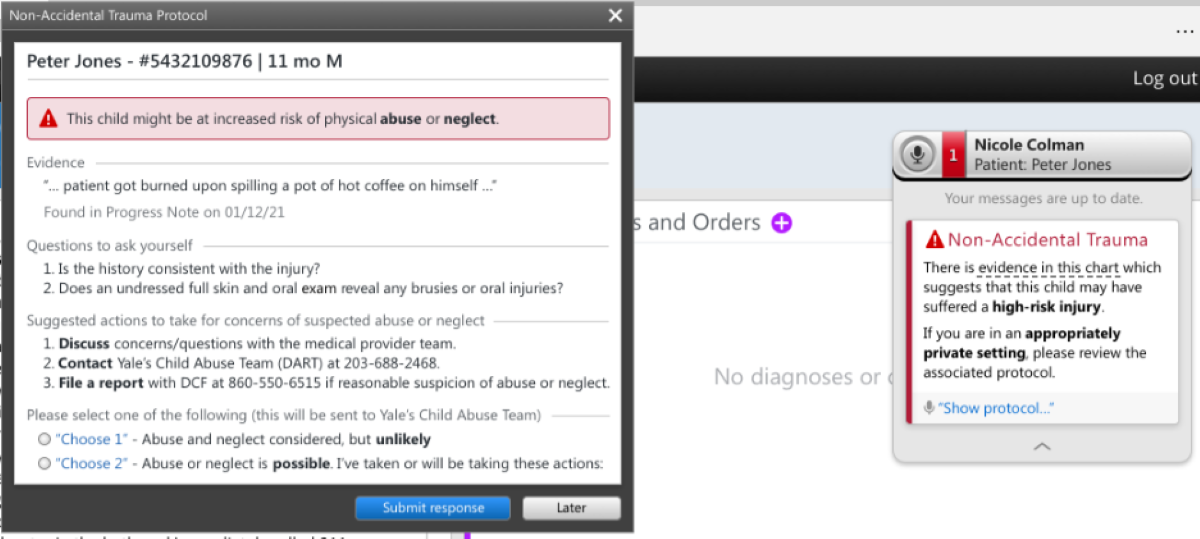
Initial first-provider version of the nursing provider–specific Child Abuse Clinical Decision Support (CA-CDS) prototype. DART: Detection, Assessment, Referral, and Treatment; DCF: Department of Children and Families; M: male.

**Figure 3:**
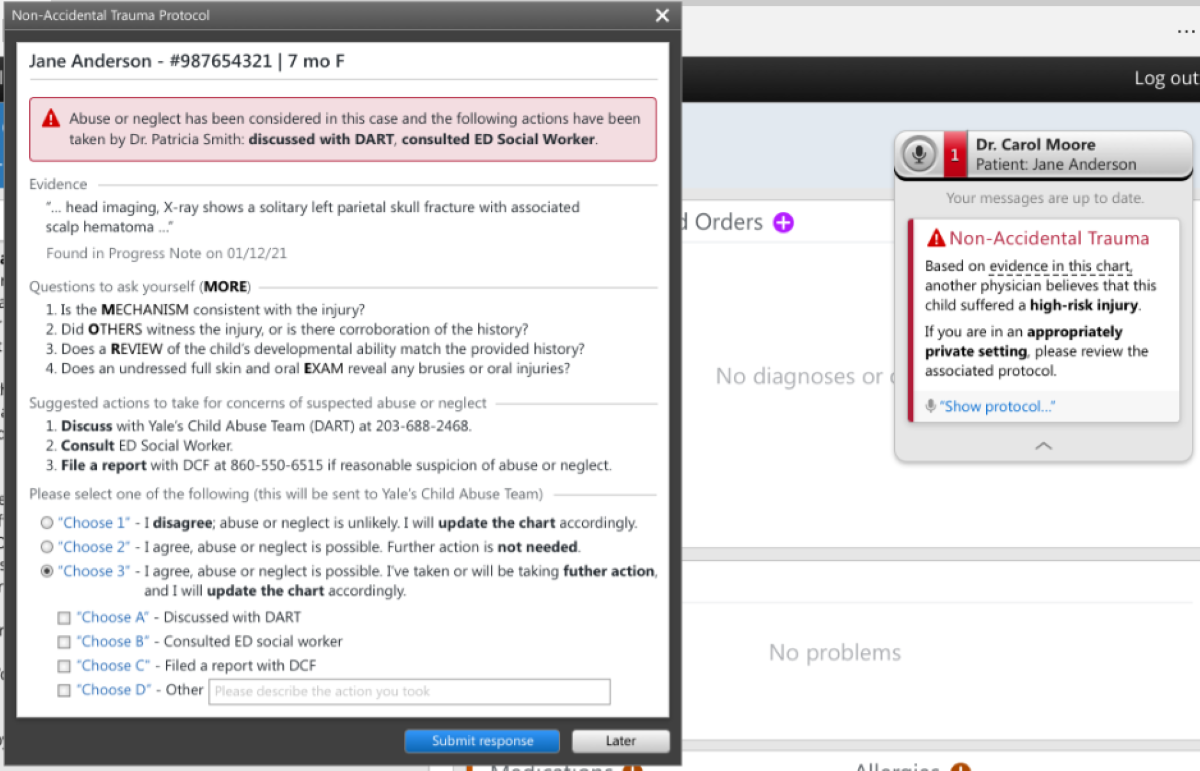
Initial subsequent-provider version of the medical provider–specific Child Abuse Clinical Decision Support (CA-CDS) prototype. DART: Detection, Assessment, Referral, and Treatment; DCF: Department of Children and Families; ED: emergency department; F: female; MORE: Mechanism, Others present, Review of development, and Examination details.

The initial CA-CDS was developed after literature review and discussions with local experts in child abuse, pediatric emergency medicine, and health informatics. The issues discussed included target users (medical and nursing providers in EDs), appropriate language, recommendations considering the local context (eg, using order sets vs consulting the local child protection team [CPT]), and degree of interruption (ie, hard-stop vs soft-stop alert in which the former requires alert completion to proceed with one’s workflow). The prototype, as depicted in Figures 1-3, consisted of a *card* and *protocol* that appeared in the EHR once the NLP algorithm identified a high-risk injury within a note’s free text. A smaller card would first appear, stating that a high-risk injury was found, with the triggering language presented in a tooltip. The card would then allow providers to open a larger protocol that presented further information about the triggering language, suggested questions for evaluation, and suggested actions for management. Users could then select between 2 acknowledgment options regarding the likelihood of child abuse or neglect and select the actions taken, which would automatically be entered into an editable documentation field. Finally, they could click *submit response* to add the documentation to the bottom of their note or *late*r to minimize the CA-CDS such that it no longer blocked the provider’s view of the EHR but remained accessible via the Fluency Direct pop-up bar. The CA-CDS was designed as a soft-stop alert such that completion was not required and workflow was not permanently interrupted.

To tailor the CA-CDS to the needs of medical versus nursing providers, the content was customized for each provider type. For instance, for medical providers (Figure 1), the suggested questions were based on the MORE (Mechanism, Others present, Review of development, and Examination details) mnemonic. The components of the mnemonic (“Mechanism: additional details about history and injury mechanism; Others present: witnesses to injury and history corroboration; Review of development: developmental ability; and Examination details: disrobed exam, specifically to examine for sentinel injuries, and additional details related to the physical examination”) aids providers in differentiating between accidental and abusive injuries [38]. For nurses (Figure 2), the content was simplified and asked whether the history was consistent with the injury. The suggested actions included recommendations to contact the local CPT (known as the Detection, Assessment, Referral, and Treatment or DART team) and file a report with Connecticut’s CPS agency known as the Department of Children and Families (DCF) as appropriate. The nursing version also recommended discussing concerns with medical providers. A subsequent-provider version was also designed to be received after another provider had already submitted their response (Figure 3). This version was equivalent to the first-provider version, except for text indicating that another provider had responded and its modified acknowledgment options, allowing the subsequent provider to disagree with the previous provider’s selection regarding the likelihood of abuse, agree without further action, or agree and take additional action.

A web-based prototype of the CA-CDS in a model EHR was designed using the InVision platform (InVisionApp Inc) for usability testing with the abovementioned features (Figures 1-3). The model EHR showed a clinical vignette of an infant presenting for emergency care for whom a provider documented a high-risk injury that triggered the CA-CDS (Table S1 in Multimedia Appendix 1). For this study, the CA-CDS was tested solely in a model EHR before future live implementation.

### Usability Testing

**Figure 4:**
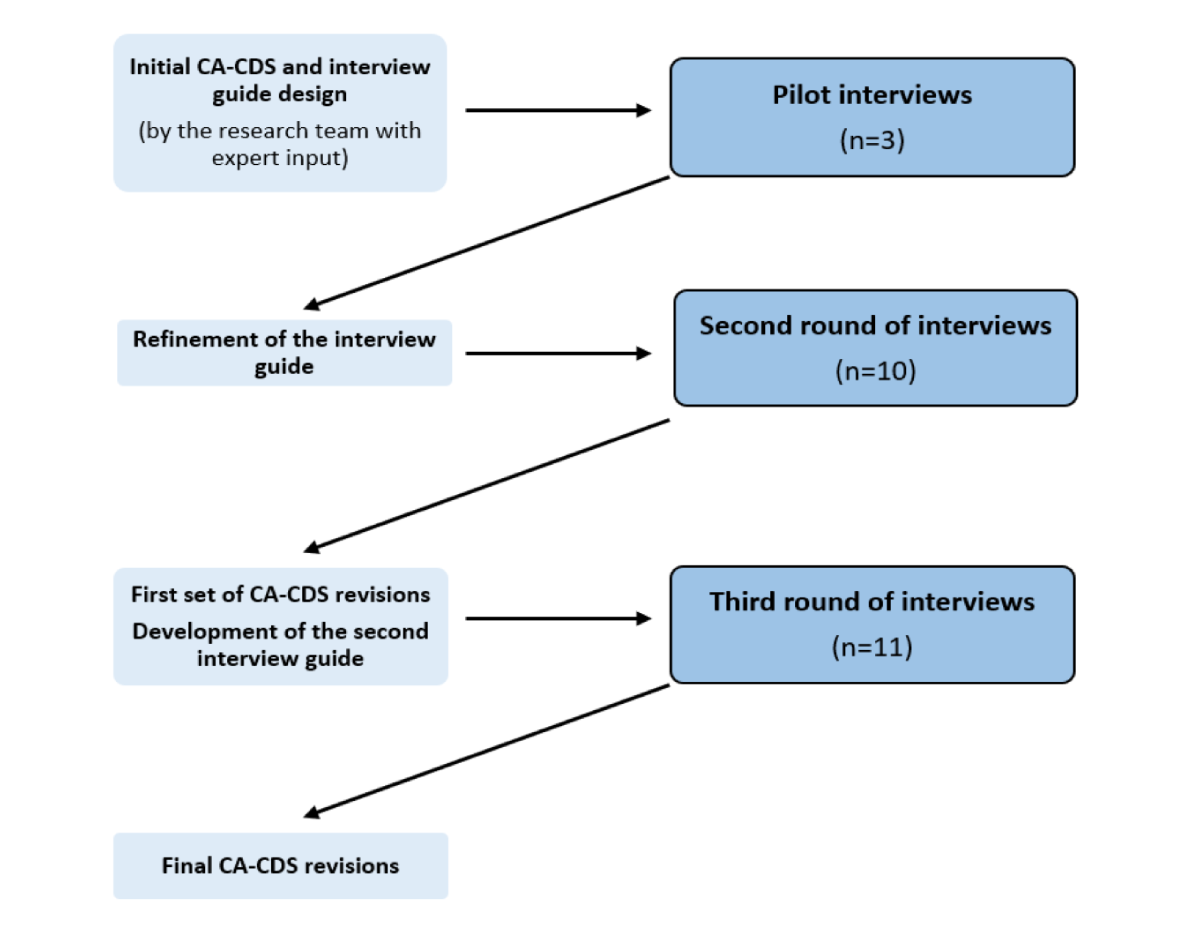
Flow diagram of usability testing. CA-CDS: Child Abuse Clinical Decision Support.

We tested the CA-CDS’s usability through a mixed methods approach. The research team, including a user design expert (KL) and researchers with qualitative research expertise (GT and AA), developed and iteratively refined an interview guide (Table S2 in Multimedia Appendix 1) with open-ended questions about topics including the CA-CDS’s design, strengths, deficits, and recommendations for improvement and future implementation. Purposive recruitment of stakeholders for interviews who represented the CA-CDS’s end users and local champions in child abuse care was conducted via email, in person, and through ED section meetings. Overall, 3 rounds of interviews were conducted by GT and AT, with audiovisual recording for documenting user-system interactions and transcript generation (Figure 4). Interviews were conducted until thematic sufficiency was achieved [39-41].

The web-based prototype (accessed via hyperlink) and interview guide were pilot-tested through in-person, think-aloud interviews with 3 ED providers (Figure 4). After further refinement of the interview guide, interviews were conducted via teleconferencing with 10 additional providers. Participants were instructed to think aloud while interacting with the prototype and then asked targeted questions using the interview guide. On the basis of the findings from the initial interviews, the CA-CDS was refined, and usability of the updated prototype was assessed with another round of interviews. Here, we sought to address topics that we felt needed more exploration such as preferred resources, documentation-related concerns, and target users for the subsequent-provider alert, and thus, we designed a more targeted interview guide (Table S2 in Multimedia Appendix 1). 11 additional ED providers were recruited in person in the ED to participate in a final round of interviews. The updated prototype was provided as a multipage PDF document on the interviewer’s tablet. The prototype was further refined based on these interviews.

Following each interview, participants were asked to complete a survey to capture demographic and quantitative usability data with adequate time and privacy for completion (Figure S1 in Multimedia Appendix 1). The survey requested the participants’ ID numbers to anonymously link their survey and interview transcript, profession and years in their role, employment site, and experience with suspected child abuse cases. To assess usability quantitatively, we used the System Usability Scale (SUS), which is a 5-point Likert scale with 10 questions exploring the different aspects of a tool’s usability and learnability. The SUS is a validated, frequently used scale that provides a quick, standardized, and easily interpretable measure for reporting and comparing a product’s usability [42,43].

### Analysis

The interview transcripts were anonymized and independently reviewed by a coding team consisting of 3 researchers, including 1 experienced in the analysis of qualitative data (GT) and 1 experienced in usability testing (KL), using conventional content analysis [44]. Team members applied codes to categorize data. Researchers then met to discuss the codes until consensus was reached, and a code list was subsequently generated and iteratively revised as new interviews were discussed [41]. The codes were then clustered into recurrent categories.

Regarding surveys, each participant’s SUS responses were scored, with the score ranging between 0 and 100 [45]. A cumulative SUS score for the CA-CDS was then obtained by calculating the median and IQR of the data set of participants’ scores. This cumulative score was compared against the curved grading scale developed by Lewis and Sauro [46] in which an SUS score of 68 corresponded to the 50th percentile of the range of scores included in their study and thus a “C” letter grade [46,47]. In their study and industry, an SUS score ≥80 indicated an above-average user experience.

### Ethical Considerations

All participants provided verbal informed consent to be interviewed and recorded before starting the interviews. Participants received no compensation. This study was approved by the Yale Human Investigations Committee (2000029566).

## Results

### Participant Characteristics

In total, 24 participants were interviewed in the study, and 23 participants completed the demographic survey. Most were physicians (13/23, 57%), from Yale New Haven Children’s Hospital (19/23, 83%), and held their current roles for >6 years (13/23, 57%; Table 1).

**Table 1:** Demographics of interviewees (n=23a).

Demographics				Values, n (%)
**Profession**				
	**Medical provider**			
		Physician assistant	2 (9)	
		Nurse practitioner	1 (4)	
		Physician (attending or fellow)	10 (43)	
		Physician (resident)	3 (13)	
	Nursing provider		7 (30)	
**Primary hospital affiliation**				
	Yale New Haven Children’s Hospital		19 (83)	
	Saint Raphael Campus		1 (4)	
	Lawrence + Memorial Hospital		2 (9)	
	Bridgeport Hospital		1 (4)	
**Duration in the role (y)**				
	<1		1 (4)	
	1-5		9 (39)	
	6-10		4 (17)	
	≥11		9 (39)	
**Average monthly exposure to cases with suspicion for child abuse (number of patients)**				
	0-1		9 (39)	
	2-5		11 (48)	
	6-10		2 (9)	
	>10		1 (4)	

^a^In total, 24 participants were interviewed, but 1 (4%) participant was unable to complete the Qualtrics survey that requested demographic data due to conflicting clinical obligations.

### Emerging Themes

#### Overview

Analysis of the interviews revealed 5 main categories of themes. These themes, along with sample subcategories and representative quotations, are presented in Table 2.

**Table 2:** Categories of themes emerging from the coding of interview transcripts.

Categories and sample subcategories		Representative quotes
**CA-CDS^a^ benefits**		
	Extra layer of protection	“The advantage is that you’re going to try to reduce the needle in the haystack phenomenon of missing these very occult cases of child abuse.”
	Inclusion of evidence-based recommendations	“I think the trigger questions are...helping us better interview patients and families, so that when we called DART^b^, we have a better history to relate to them...It’s helpful that there’s phone numbers and...that it kind of prompts you through the next steps.”
	Alerting the entire clinical ED^c^ team	“It gives each individual provider a chance to document what they need to document about their concerns for each individual patient.”
**User-centered, workflow-compatible design**		
	Customization based on provider type	“From a nursing end, obviously discussing it with the care team, with all of us being involved and voicing our concerns on the child.”
	Soft-stop alert configuration	“Hard stops...could be in the wrong place...I think soft is good. I think being able to close [the alert] because [the flow] is just so unpredictable.”
	Editable and automatic documentation	“I think as long as those options can be documented in the chart just to save somebody the step of writing...That would be helpful.”
	Triggers from multiple providers’ notes	“You getting to the point of actually doing documentation is midway or further down. So, if it’s also looking at the nursing notes..., then on [opening the] chart, I would get this message.”
	Clear presentation of alert trigger	“[The evidence] is factual...They can get everybody on the same page with what the concern is, and what everybody needs to be aware of that’s caring for that child.”
	Attention-grabbing design elements	“I like that it has...the exclamation point with the red triangle that, kind of, alerts you to pay attention to it.”
	Accessible recording of the previous provider’s actions	“I think [the previous provider’s actions box] is good because that’s the sign-out. So, I think that’s reiterated as what should be signed out from the prior provider.”
**Recommendations for improvement**		
	Consolidating the content	“As opposed to having so much in this ‘Please select one of the following, this will be sent to the Yale child abuse team’ stuff popping up, [I wonder] if the suggested actions for concern of suspected abuse and neglect...could be clickable themselves.”
	Clearer design elements	“I don’t know if [the evidence tooltip] was a different color or, like, you know like a hyperlink is in an email...how it shows up as a different color and underlined...I just wasn’t aware that’s what it was representing.”
	Adding a hyperlink to additional resources	“The diagnosis is a fracture. Then here, for all this stuff, there can be direct links whether it’s either mandated, like happens automatically, or they can link to it to see what the evidence actually is.”
	Adding further information about the trigger source	“But if it’s not your note, is there a way to more readily send to the specific note?...A progress note, that’s pretty generic...but it might be nice if there’s a way to get a little more specific, that it was the triage note or.”
	Modifications to better reflect the provider workflow	“I submit that, then this just completely disappearing...it would seem to me, maybe having it be completely disappeared, and if you want, at the point of discharge, to maybe one more time give me the opportunity to say, ‘Are you really? Just think about this one more time’.”
**Barriers to future implementation**		
	Alert fatigue	“The drawback is that you’ll...probably have to trigger 10 alerts for every kid that is a true positive...So, there might be fatigue with the alert.”
	Infringement of provider autonomy	“There are some sentiments that this is being forced on somebody without any evidence...I don’t want to call them naysayers, but they don’t really believe the bibliographic evidence.”
	Hesitancy to change	“It seems like every week we’re getting told to do something new. So honestly, I really feel like as much as people don’t like change, we’re just getting told this is the way it is, and you need to do it.”
	Concerns regarding documentation	“If I am going to contact DCF^d^, and my husband’s the one that’s beating my kid, and now it’s available per the Cures Act, they know that DCF was contacted. You might be putting me and my kid at risk...Just putting it in the notes...[which] are being released to everybody now who has access, that could be a safety concern.”
**Facilitators of future implementation**		
	Stakeholder buy-in	“To communicate with physicians, practitioners, you need to do it five different ways. So, I think probably asking to come to staff meetings...that system ED leadership committee...to do high-level show-and-tell.”
	Provider education	“I think as long as there is some kind of super user that can educate everybody on it...It’s a matter of just getting shown once how to do something, and then it kind of sticks.”
	Sharing the system’s test characteristics	“I would love to have some evidence to show that that's benefiting somebody, hopefully the patient...Just some retrospective data to show the veracity or the utility of your system, however you define it, either accuracy or times that you picked up something that the physicians should have or didn’t.”

^a^CA-CDS: Child Abuse Clinical Decision Support.

^b^DART: Detection, Assessment, Referral, and Treatment.

^c^ED: emergency department.

^d^DCF: Department of Children and Families.

#### CA-CDS Benefits

Participants discussed the challenges of recognizing abusive injuries, especially those that were subtle or “minor.” They expressed that the CA-CDS could provide an extra layer of protection against missing abuse by reminding providers in real time to consider abuse in their differential diagnosis. Users also appreciated the CA-CDS’s evidence-based recommendations for evaluation and management that included guidance about important historical information to be collected and about using the expertise of specialists. Specifically, they found the MORE mnemonic to be clear, memorable, and helpful to improve information gathering, decision-making, and documentation. Participants also valued the emphasis on consulting specialists to determine the appropriate workup as it enabled the CA-CDS to remain simple but adaptable despite case-specific variations. In addition, the users appreciated that the CA-CDS alerted both medical and nursing providers, allowing for open communication of concerns among the entire clinical team.

#### User-Centered, Workflow-Compatible Design

Participants discussed the elements of the CA-CDS that would optimize their workflow. First, they valued the CA-CDS’s customization based on provider type, which reflected workflow differences, and preferred that nurses and medical providers submit independent CA-CDS responses. For instance, nurses favored the recommendation to discuss concerns with the medical team rather than consulting the CPT directly as it reflected the typical nursing workflow. Second, users felt that the CA-CDS’s soft-stop alert configuration, which could be minimized and reaccessed on demand, would be more flexible around providers’ unpredictable workflow. Third, users expressed that the documentation component, which automatically populated the selected actions into the note while also remaining editable for providers to share their own decision-making, would avoid redundancy. Fourth, participants appreciated that the CA-CDS could be triggered by injuries in various providers’ notes. In particular, they expressed that by including nursing notes, which are often created before other clinicians’ notes, as a trigger source, the CA-CDS could allow for more timely evaluation. Fifth, users shared that having the alert explicitly identify the triggering documentation enabled all team members to quickly be on the same page regarding the specific injury causing concern for abuse and allowed providers to assess if their documentation was being construed as intended. Sixth, participants appreciated the CA-CDS’s attention-grabbing formatting. Features such as bold text, colorful symbols, and high-risk injury phrasing helped emphasize the alert’s significance. Finally, regarding the subsequent-provider CA-CDS version, participants valued that the protocol clearly displayed the actions selected by the provider who initially submitted a response. They found this helpful for handoffs, highlighting the salient concerns and workup for all team members.

#### Recommendations for Improvement

Users made several recommendations to improve the CA-CDS’s usability. Participants recommended consolidating the content to reduce information overload. For instance, they suggested removing the acknowledgment section to reduce redundant text, allow more flexibility for documentation, and circumvent the potential legal implications of documenting disagreement with previous providers. To improve clarity, users also suggested using obvious underlining and bold colors to highlight design elements such as hyperlinks and editable text fields. In addition, to improve providers’ case-specific and general knowledge about abuse, participants suggested adding a link to additional resources that providers could access for further support.

Next, users requested further information about the source of the triggering documentation, including its author and location, to better find and assess the triggering content. Finally, providers suggested modifications to improve their workflow and use of the CA-CDS. For example, nurses appreciated the card’s reminder to be in an appropriately private setting as they often charted near patients, whereas medical providers supported removing this component which was less relevant to their workflow. They also shared that the reappearance of the CA-CDS at discharge could serve as a reminder to those who had missed, ignored, or initially not felt ready to complete the alert and as an opportunity to add more information and reconsider abuse in their differential.

#### Barriers to Future Implementation

First, participants warned about the potential for alert fatigue, especially if there were several false positives or if excessive effort was required for completion. They discussed how alert fatigue may lead providers to ignore the alert or seek work-arounds such as documenting in a manner to avoid triggering the alert. Second, they shared that the CA-CDS, with its interruptive alert and recommendations to consult specialists, may be perceived by some providers as infringing on their autonomy. Third, users counseled that providers may be accustomed to a particular manner of providing care and hesitant to change.

Finally, users warned that providers may be wary of using the CA-CDS, especially its documentation component, given the potential consequences of documenting concerns about abuse in notes that are accessible to caregivers. These included liability, inadequate patient-sensitive language, caregivers learning about concerns before discussions with the medical team, and caregivers purposefully obstructing care or inflicting further harm. However, users also responded that they tried to document objectively, being mindful about how their documentation could be interpreted by caregivers. On the basis of these discussions, the following text was added to the protocol: “Consider ‘unsharing’ the note ‘to prevent substantive harm to patient or another person.’”

#### Facilitators of Future Implementation

Participants recommended several strategies to optimize the CA-CDS’s implementation. Users felt that stakeholder and leadership buy-in and support for the system would promote future use and sustainability. In addition, participants stated the importance of educating providers regarding how to use the system to appropriately manage the cases of potential abuse and how to approach caregivers based on evidence-based recommendations. Users also recommended communicating the accuracy of the CA-CDS and its triggering NLP algorithm by sharing the system’s validation data and instances where the system could have made a difference.

### Prototype Revisions

**Figure 5:**
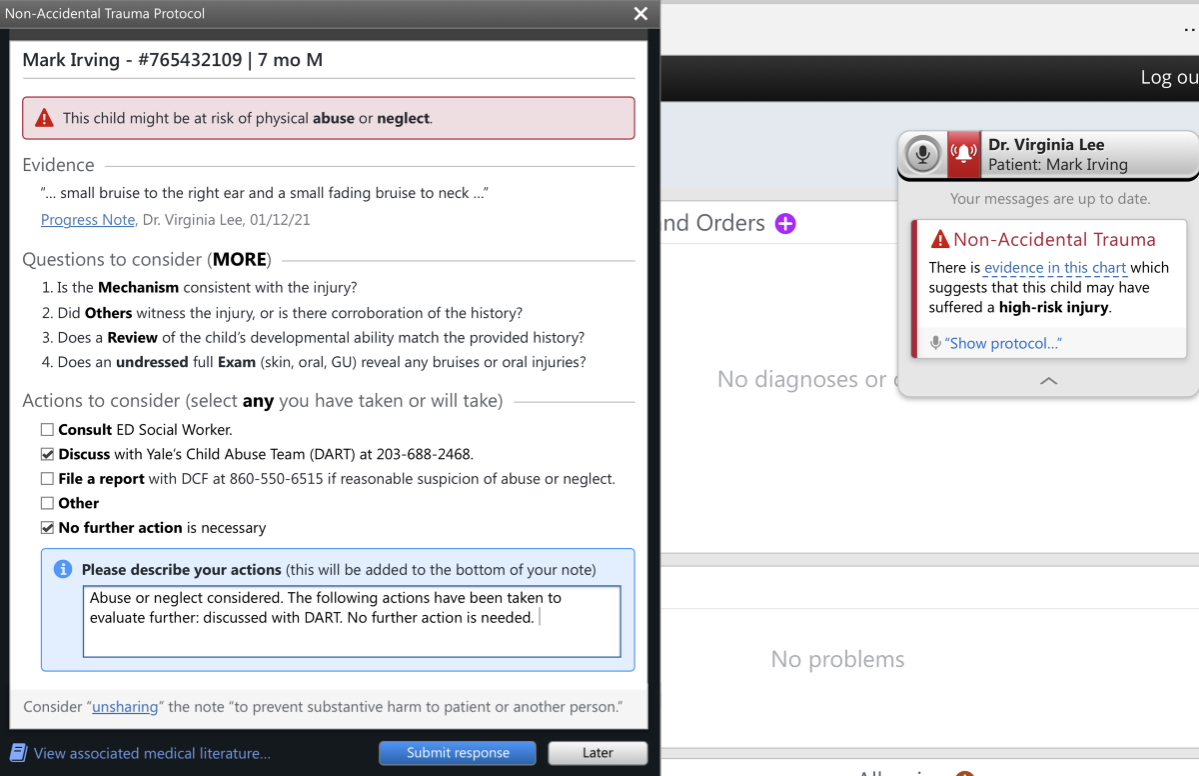
Final prototype of the first-provider version of the medical provider–specific Child Abuse Clinical Decision Support (CA-CDS). DART: Detection, Assessment, Referral, and Treatment; DCF: Department of Children and Families; ED: emergency department; M: male; MORE: Mechanism, Others present, Review of development, and Examination details.

**Figure 6:**
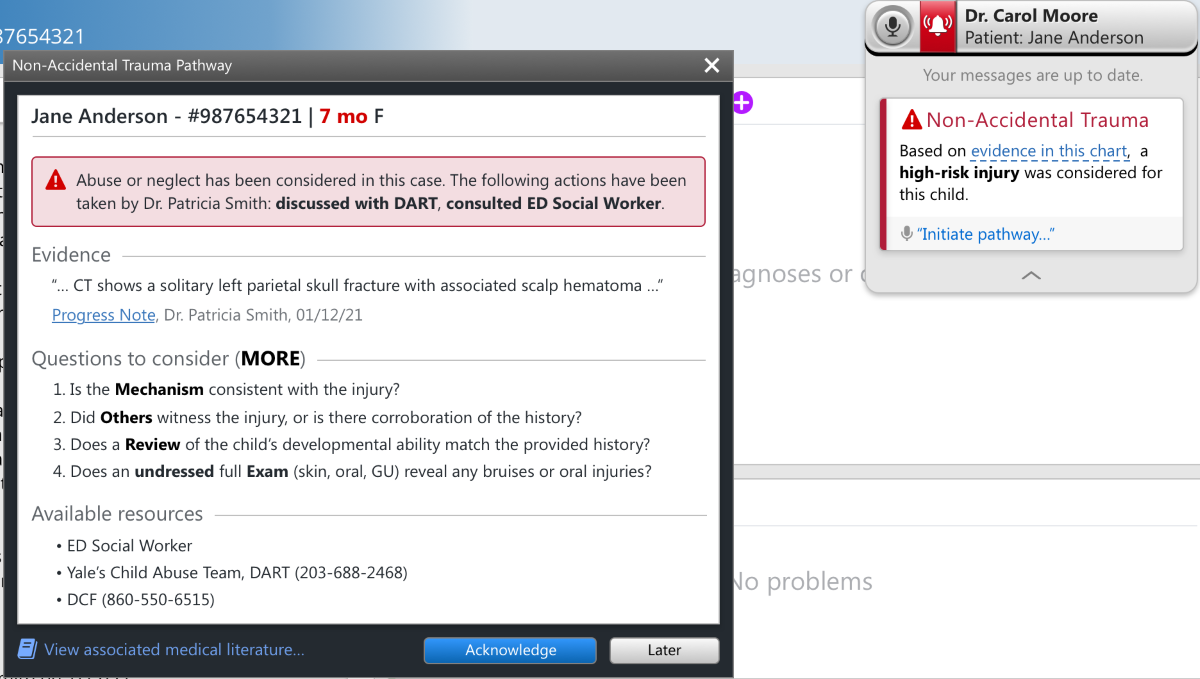
Final prototype of the subsequent-provider version of the medical provider–specific Child Abuse Clinical Decision Support (CA-CDS). DART: Detection, Assessment, Referral, and Treatment; DCF: Department of Children and Families; ED: emergency department; F: female; MORE: Mechanism, Others present, Review of development, and Examination details.

On the basis of the interviews and feedback from our team of experts, multiple rounds of modifications were performed to create our final prototype (Figures 5 and 6). All modifications are listed in Table S3 in Multimedia Appendix 1.

### SUS Scores

Of the 24 interviewees, 23 (96%) completed the SUS. Scores ranged from 62.5 to 100, with a median of 80 (IQR 75-92.5). Compared with Lewis and Sauro’s [46,47] curved grading scale, our median corresponded to the 85th to 89th percentile and an A− letter grade.

## Discussion

### Principal Findings

Usability testing of the CA-CDS revealed several key findings. Users valued the additional protection against missing abuse that is offered by the alert to the entire clinical team and the presence of evidence-based recommendations for the evaluation and management of suspected abuse. Users also appreciated the CA-CDS’s user-centered, workflow-compatible design elements that captured the user’s attention to provide timely, provider-specific information while minimizing interruptions and redundancy. However, they recommended improving the system’s clarity and brevity, highlighting critical features such as the triggering documentation’s source, and further supporting the users by offering additional resources and alert reappearance at discharge. User recommendations informed the iterative refinements of the CA-CDS prototype. Future studies will be directed toward the implementation and live testing of the revised, user-centric CA-CDS within our hospital system’s EHR.

### Comparing the CA-CDS With Existing Systems

Researchers have described the development, implementation, and evaluation of a child abuse CDS system for pediatric and general EDs that identified high-risk injuries through a variety of alert triggers including specific screening results, orders, and discharge documentation [26-31]. Their CDS system notified providers about the concern for abuse and recommended direct connection to an age-appropriate, injury-specific order set or a CPS referral. While the CA-CDS similarly aimed to identify high-risk injuries and provide CDS regarding the evaluation of suspected abuse, there were numerous differences. The current CA-CDS was triggered via an NLP algorithm that examined all the free text in the notes of medical providers, nursing providers, and SWs, whereas previous CDS systems were triggered primarily by discrete fields, active screening such as those completed by nurses upon evaluation, or limited NLP function that could only examine the free text within the chief complaint and focused assessment fields [33]. An entirely NLP-triggered CDS system may allow for minimal interruptions to the workflow; be more acceptable to frontline providers; and allow the CA-CDS to be triggered as soon as there is any documentation, even as early as triage, without requiring actions outside the normal workflow.

While many existing CDS systems connect users to standardized order sets [26-31] and recent consensus guidelines also recommended the use of a physical abuse order set with consistent and evidence-based actions [14], most of our users (13/24, 54%) preferred simpler suggested actions with reminders to consult a SW or the CPT to aid in nuanced decision-making. Consultation with these specialists may facilitate appropriate decision-making around performing additional testing or reporting to CPS and reduce bias in evaluation and reporting of suspected child abuse [48,49]. However, to acknowledge the importance of autonomy for users, the CA-CDS was designed as a nonmandatory, soft-stop alert that included a free-text response option and a hyperlink to additional resources including local clinical pathway guidelines to provide either support or an avenue for independent decision-making depending on the provider’s needs. Next steps include comparing the outcomes of systems that recommend standardized order sets to those that recommend consulting clinicians.

Finally, the CA-CDS was hosted on external software from 3M Company and designed to be subsequently integrated into the EHR, rather than being directly built into the EHR. This design aligns with the Fast Healthcare Interoperability Resources (FHIR) data standard that standardizes how information is stored, used, and exchanged between computer systems and thereby streamlines software development to support health care needs [50,51]. EHRs with FHIR-enabled technology allow for the packaging of information from the EHR into discrete, standardized units that can be interpreted and acted upon by external applications including CDS systems. FHIR-based applications, such as those used in this study, allow the results of a CDS system to trigger the opening of order sets or to directly provide CDS within the EHR and may realistically solve the problem of 1 child abuse CDS system communicating with multiple EHRs [31,52,53]. With 84% of hospitals in the United States having adopted FHIR-enabled technology and 3M’s connection with hundreds of EHR systems [51,54,55], the CA-CDS’s FHIR-based application design may facilitate the system’s dissemination across numerous institutions and EHRs.

### Examining the Rigor of the CA-CDS

Consistent with the recommendations for successful, guideline-based, computerized CDS as described by the GUIDES checklist and child abuse expert consensus recommendations [14,22,37], the CA-CDS was developed by a team of local experts to provide evidence-based guidelines for the evaluation and management of high-risk injuries, reflective of recent studies in the field. In addition, the CA-CDS was integrated with an objective and internally validated NLP algorithm that captured data widely in the notes of ED SWs, nursing providers, and medical providers [33]. Given that the system is triggered independent of the providers’ gestalt and background, the CA-CDS may improve the standardization of patient care and reduce the impact of providers’ implicit biases [49,56].

The CA-CDS met the recommended design standards in several ways. Users’ feedback demonstrated the system’s usability, with users finding the CA-CDS to be user friendly, concise, and clear. The CA-CDS was intentionally refined based on feedback to reflect the users’ preferences. Considerable effort was made to integrate the system into providers’ clinical and EHR workflow to minimize interruptions and redundancy. The CA-CDS also provided ample flexibility around decision-making through features such as editable and automatic documentation and soft-stop alert design. Interestingly, in contrast to experts’ recommendation to incorporate automated referrals, standardized CPS reporting, and a multidisciplinary audience [14,22], most users preferred to keep the CA-CDS simple without these features (19/24, 79%) and limit the alert’s recipients to the primary clinical team (7/13, 54%). Next steps include examining the real-time use of the CA-CDS by ED clinicians.

Similar to the consensus recommendations, participants discussed the importance of planning for future implementation [14,37]. They identified the facilitators of future implementation, such as stakeholder buy-in, education about the CA-CDS and the accuracy of the underlying trigger (ie, the NLP algorithm), and iterative refinement of the system based on user feedback. While participants discussed alert fatigue, or provider desensitization owing to excessive alerts [57], as a potential barrier to future implementation, the NLP algorithm’s relatively high specificity and the limited patient population may minimize this concern. However, continual improvement of any rule-based algorithm is critical to maintain its quality. While participants did not discuss the implications of receiving a CA-CDS alert after a patient’s discharge if a provider completes the documentation after a patient’s ED visit, encounters with injuries identified by the NLP algorithm undergo weekly routine case surveillance by the CPT [58]. This is especially important for cases in which documentation is completed after a patient’s discharge to assure that the identified injuries are not concerning for missed abuse. Such a monitoring system may facilitate the identification of cases that might have been missed in real time during the ED encounter [14].

### Patient Access to Electronic Health Information

An important but underexplored aspect of child abuse CDS systems is the impact of the 21st Century Cures Act on providers’ EHR interactions. The Cures Act is a federal law that came into effect in April 2021, mandating the free, timely release of electronic health information to patients and their guardians unless the practice meets the condition of select exceptions, one of which is preventing harm in contexts such as child abuse [59-61]. Concurrently, adoption and use of the patient portal dramatically increased as a result of the COVID-19 pandemic, with much of patient care and communication shifting to electronic mediums. Given the increased ease of patient access to EHR content, it is especially important to understand how provider perspectives about documentation of suspected child abuse have been affected by the new law. This study was uniquely timed to explore these concerns following the Cures Act. Although users worried about the potential repercussions of using the CA-CDS to document suspicions about abuse in caregiver-accessible notes, their unease was alleviated by the clarity provided by the protocol regarding the destination of the automatic documentation into the note and the addition of a reminder to unshare the note if appropriate. Next steps include exploring caregivers’ perspectives about the documentation of suspected child abuse.

### Limitations

This study had at least 3 limitations. First, although we tried to recruit representative participants, few community ED providers (4/23, 17%) versus pediatric ED providers (19/23, 83%) participated in the usability testing. As providers who work at sites that often see most of a community’s pediatric population and more often underdiagnose child abuse [6-8], feedback from community ED clinicians is uniquely valuable. Future system testing would benefit from having a more balanced or community-focused participant pool. Second, we modified the CA-CDS based on the majority’s preferences. As such, there may have been modifications desired by a notable percentage of our users that were not implemented, which may limit their interaction with the CA-CDS in the future. However, the high-risk injuries identified by the CA-CDS will be routinely reviewed by the CPT to assure that cases are not misdiagnosed. Third, while this system was developed by a local team of experts and through iterative usability testing with providers at different sites, the CA-CDS’s recommended management may be institution specific. Further usability testing may be required if the system is disseminated to other hospitals, especially those with limited resources.

### Conclusions

In summary, with its user-centered design and evidence-based content, the CA-CDS offers a novel method to aid ED medical and nursing providers in the real-time recognition, evaluation, and management of infant physical abuse. Our system has the potential to reduce the number of missed cases and increase the provision of less biased and evidence-based care to all infants.

## Appendix

Multimedia Appendix 1 Supplementary tables and figures including clinical vignettes, interview guide, demographic and System Usability Scale survey, and prototype modifications.

## Abbreviations

CA-CDS: Child Abuse Clinical Decision Support
CDS: clinical decision support
CPS: Child Protective Services
CPT: child protection team
DART: Detection, Assessment, Referral, and Treatment
DCF: Department of Children and Families
ED: emergency department
EHR: electronic health record
FHIR: Fast Healthcare Interoperability Resources
GUIDES: Guideline Implementation with Decision Support
MORE: Mechanism, Others present, Review of development, and Examination details
NLP: natural language processing
SUS: System Usability Scale
SW: social worker

## Data Availability

The data underlying this paper will be shared upon reasonable request to the corresponding author. The technical components used in this study were provided by 3M Company under license and will be shared upon request to the corresponding author with permission from 3M Company.
