# Correction: Developing and Testing the Usability of a Novel Child Abuse Clinical Decision Support System: Mixed Methods Study

**DOI:** 10.2196/60444

**Published:** 2024-05-27

**Authors:** Amy Thomas, Andrea Asnes, Kyle Libby, Allen Hsiao, Gunjan Tiyyagura

**Affiliations:** 1 Department of Pediatrics Yale University School of Medicine New Haven, CT United States; 2 3M | M*Modal 3M Health Information Systems 3M Company Maplewood, MN United States

In “Developing and Testing the Usability of a Novel Child Abuse Clinical Decision Support System: Mixed Methods Study” (J Med Internet Res 2024;26:e51058), the authors noted one error.

The "Background" section of the abstract was originally published as:

Despite the impact of physical abuse on children, it is often underdiagnosed, especially among children evaluated in general emergency departments (EDs) and those belonging to racial or ethnic minority groups. Electronic clinical decision support (CDS) can improve the recognition of child physical abuse.

It will now be changed to:

Despite the impact of physical abuse on children, it is often underdiagnosed, especially among children evaluated in emergency departments (EDs). Electronic clinical decision support (CDS) can improve the recognition of child physical abuse.

The correction will appear in the online version of the paper on the JMIR Publications website on May 27, 2024 together with the publication of this correction notice. Because this was made after submission to PubMed, PubMed Central, and other full-text repositories, the corrected article has also been resubmitted to those repositories.

