# Supplementary material for: Developing and Testing the Usability of a Novel Child Abuse Clinical Decision Support System: Mixed Methods Study
Source: J Med Internet Res. 2024 Mar 29;26:e51058. doi: 10.2196/51058 (PMC11015363; doi:10.2196/51058)
Supplement: Multimedia Appendix 1 [file jmir_v26i1e51058_app1.docx]

**Multimedia Appendix 1: Supplementary Tables and Figures**

**Table S1.** Clinical vignettes for model electronic health record.

| Mark Vignette  (7-month-old male) | Mark is brought into the Emergency Department by his mother. She states that he has been particularly gassy and irritable since his morning feeds. As such, she’s been comforting and tending to him constantly for the last few hours. About an hour ago, she felt dizzy and accidentally tripped, falling forward to the floor, while still holding Mark to her chest.  Vital signs are stable. On physical exam, the child is well-appearing and interactive, babbling in the room while his mother is trying to make a bottle. Exam is only notable for a small bruise to the right ear and a small fading bruise to neck. Labs are unremarkable.  Father reaches the waiting room an hour after the patient and his mother. He confirms that the child has been gassy and irritable all day and states that his wife tripped and fell against the wall. Both he and his wife are glad to hear that Mark only has minor bruising and thinks that it was likely from the fall. |
| --- | --- |
| Jane Vignette  (7-month-old female) | Jane is brought to the Emergency Department by her worried mother after rolling off the diaper changing table and falling to the floor this evening when her mother had turned away for a moment to get a new diaper. Her mother states that the changing table is about 3 feet off the ground and that she turned back around just as Jane hit the floor and started crying. She is not sure which parts of the Jane’s body hit the floor on impact.  Vitals are stable. Physical exam shows an irritable child with some left scalp edema and a hematoma over the left parietal region. On pursuing head imaging, X-ray shows a solitary left parietal skull fracture with associated scalp hematoma. Labs are unremarkable. |
| Peter Vignette  (11-month-old male) | Peter is brought into the Emergency Department by ambulance with his mother who is very upset. Ambulance staff tells you that the mother called them after the patient got burned upon spilling a pot of hot coffee on himself. When you ask the mother for more details, she tells you that he had been playing in the kitchen while she was making breakfast this morning. Her 2 older children started fighting in the living room, and while she was there with them, she heard some glass shattering and Peter crying from the kitchen. In rushing to the kitchen, she found Peter sitting on the floor crying, with his upper chest quickly getting redder and more swollen, and the shattered coffee pot from the table next to him. She quickly washed him with cold water in the bath and immediately called 911. |

**Table S2.** Semi-structured interview guide.

| **Interview Questions^a^** | | |
| --- | --- | --- |
| 1. Please share your thoughts on the look and language of the CA-CDS^b^ card? | | |
| 1. Please share your thoughts on the positioning of the CA-CDS card within your Epic screen? | | |
| 1. If you were to see a card like this pop up on opening the chart or while writing the note, please describe your reaction? | | |
|  | | - 1. What do you think caused this alert to appear? |
| 1. If this CA-CDS alert card were to pop up in a patient room, what concerns, if at all, would you have? | | |
|  | | - 1. Is title of Non-Accidental Trauma and language of card appropriately patient-sensitive? If not, what can we change? |
|  | | - 1. *If participant mentions minimizing or closing the card*—What would be the best way to go about that? |
| 1. Please share your thoughts on the look, content, and language of the protocol? | | |
|  | - 1. *If participant requests extra action options for the acknowledgements section*—Tell us more about what would be the most convenient way for us to provide that? | |
| 1. In the typical clinical setting, please describe why you might click later instead of the submit response button? | | |
| 1. How would you use the free text option, if at all, in this clinical scenario? | | |
|  | - 1. What are the advantages vs. disadvantages of having free text? | |
|  | - 1. The current design has free text as one of the options for submission. With this setup, would you be concerned about a workaround? Why? | |
| 1. This CA-CDS suggests actions for providers to take while evaluating for child abuse. Other CDS^c^ tools have included order sets and other content. What would be your preferred content to have in a CDS for child abuse? Why? | | |
| 1. How do you feel about having documentation in the EHR^d^ that this CA-CDS was triggered? | | |
|  | - 1. What are your thoughts on the language? | |
| 1. What is the ideal destination for this documentation? | | |
|  | - 1. Should this go into the note, elsewhere in the chart, and/or somewhere other than the chart? | |
| 1. This CA-CDS is currently designed to be a “soft” alert such that its completion is not required for you to close the encounter though the smaller card will always be accessible once triggered. What are the advantages versus drawbacks of a system like this? | | |
|  | - 1. Can you envision and share any scenarios where you might look for a workaround or ignore this CA-CDS? | |
|  | - 1. What would you change, if anything, to assure providers like yourself avoid these workarounds and provide a response to the protocol? | |
| 1. Currently, this CA-CDS is only designed to trigger for medical and nursing providers in the ED^e^. Should anyone else also receive this? Why? | | |
| 1. Please share your thoughts on the look and the language of the subsequent provider version of the card and protocol? | | |
| 1. Would you feel comfortable disagreeing and/or adding actions? | | |
| 1. Describe the use, if at all, of having this alert continue to pop up for each provider independent of whether another provider has submitted their response? | | |
|  | - 1. Does this rationale change if the previous provider was a nursing provider versus a medical provider? | |
| 1. With everything you’ve seen so far, tell me your overall feelings about this CA-CDS prototype? | | |
|  | - 1. What are some benefits to this CDS system and what are some drawbacks? | |
| 1. If you could change one thing about the CA-CDS prototype that you just saw, what would that be? | | |
|  | - 1. Do you have any suggestions to increase the acceptance of this software in the ED? | |
| 1. Can you think of any barriers that we might run into while trying to implement this software in the ED? | | |
| 1. Should anything be modified in the first provider version of the card or protocol to be more useful to providers? | | |
| 1. On the topic of resources, what are your feelings on linking this protocol to an order set that includes common orders in the evaluation of suspected child abuse? | | |
|  | - 1. Are there any other resources that would be useful to you? | |
| 1. *If participant is a nursing provider*—What do you think about the appropriately private language? | | |
| 1. Our CDS is presented as a soft alert such that a provider could ignore and not interact with the CDS and continue their workflow without interruption. Would having the card reappear at discharge increase the likelihood of your interaction with and completion of the protocol? | | |
| 1. What are your thoughts on the documentation field provided in this protocol? | | |
|  | - 1. How, if at all, would you change this? | |
| 1. How, if at all, has the Cures Act, which allows for more real-time release of patient records to families, impacted your documentation on cases of suspected abuse? | | |
| 1. Did you notice the “View associated medical literature…” link? What would you expect to see after clicking it? | | |
| 1. How do you feel about the language in the subsequent provider card? | | |
| 1. During a patient’s ED stay, there may be several medical or nursing ED providers that evaluate the child. After a provider submits a response to this alert as the “first provider”, in your opinion, how many providers and which providers (eg. the attending?) should receive and acknowledge this “subsequent provider” alert? (i.e. just one provider after the first submitter or a certain number of providers or every provider present while the patient is in the ED)? | | |

- 1. Questions 1-18 were utilized in the first two rounds of interviews. Questions 19-27 were utilized in the third round of interviews.
  2. CA-CDS: Child Abuse Clinical Decision Support.
  3. CDS: clinical decision support
  4. EHR: electronic health record
  5. ED: emergency department


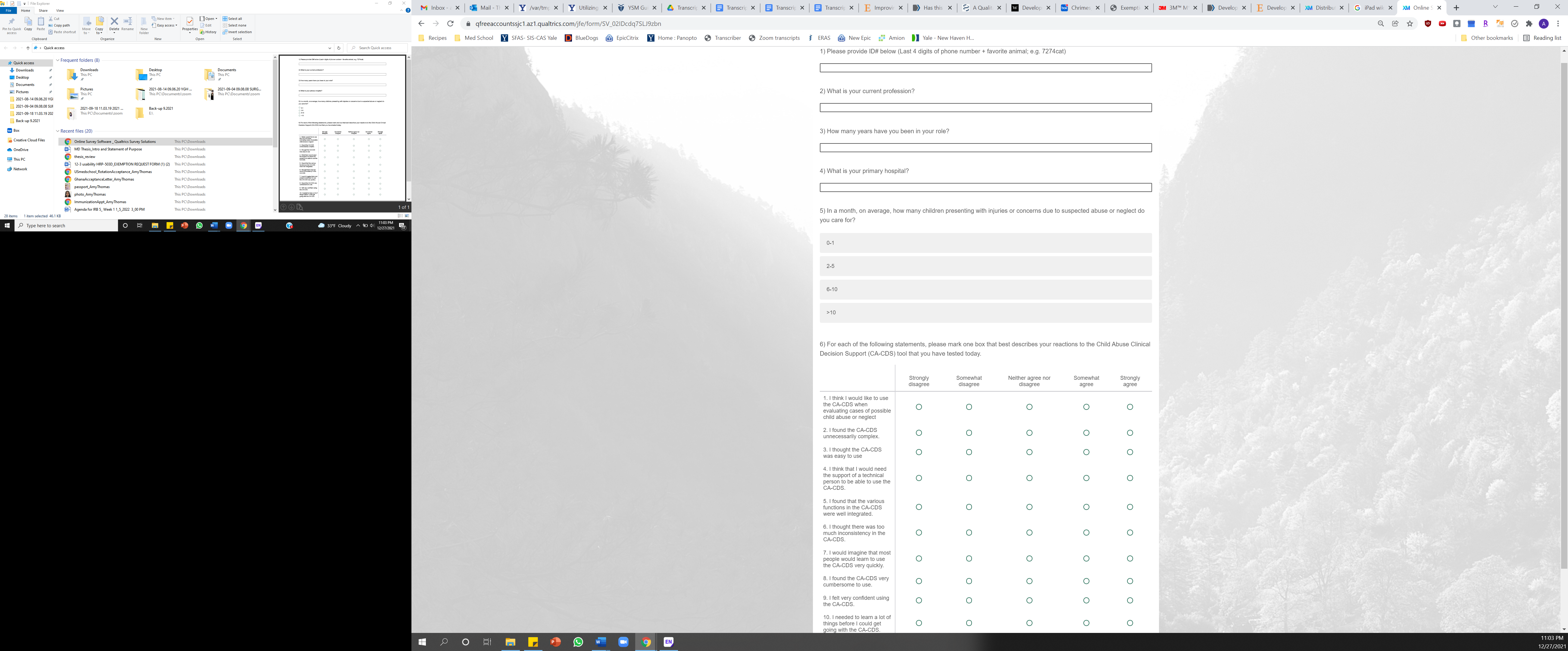


**Figure S1.** Survey for demographic data collection and System Usability Scale.

**Table S3.** Modifications to prototype based on user feedback.

| **Recommendations for Improvement** | **Modifications to Prototype** |
| --- | --- |
| 1. Consolidating text | 1. Modified “Questions to Ask Yourself” heading to “Questions to Consider”^a^ 2. Protocol’s red box text modified from “…might be at increased risk…” to “…might be at risk…”^b^ 3. Modified “Suggested Actions…” heading to “Actions to Consider (select any you have taken or will take)” and added clickable checkboxes to each action^b^ 4. Added “Other” and “No further action is necessary” as additional action options under “Actions to Consider”^b^ 5. Removed free text box associated with Other choice in acknowledgement options and consolidated with editable documentation box^b^ 6. Removed acknowledgement of likelihood component and modified automatic text populating into the editable documentation box to “Abuse or neglect considered” to reflect this^b^ 7. Removed diagnosis agreement/disagreement component and replaced “Submit Response” button with “Acknowledge”^c^ 8. Replaced portion of first paragraph of card that stated “another [physician/nurse] believes that this child suffered a high-risk injury” to “a high-risk injury was considered for this child”^c^ 9. Replaced “Suggested actions to take…” heading with “Available Resources” and simplified content in this section to name and contact information of resource as applicable^c^ 10. Removed second paragraph of card (recommending appropriately private setting) and replaced with “You’re about to see sensitive information. Once you’re ready, initiate the pathway below.”^d,e^ |
| 2. Clearer design elements | 1. Replaced alert notification number in Fluency Direct pop-up bar with a bell icon^a^ 2. Changed color of evidence tooltip in card to blue to make it clearer that it can show additional information^a^ 3. Changed color of patient age in protocol to red to draw more attention^a^ 4. Bolded whole word that made up MORE acronym^f^ in each question rather than just the first letter of each word^a^ 5. Updated design of editable documentation box to only appear after selection of an action for ease of using correctly^b^ 6. Modified header for documentation box from “The following text will be added to the bottom of your note” to “Please describe your actions (this will be added to the bottom of your note)”^b^ 7. Added book icon next to “View associated medical literature” hyperlink^a,d^ 8. Replaced “Show protocol” with “Initiate pathway” and renamed “Non-Accidental Trauma Protocol” title of protocol box to “Non-Accidental Trauma Pathway” to emphasize its interactive, soft-stop design^a,d^ |
| 3. Adding a hyperlink to additional resources | 1. Added “View associated medical literature” hyperlink to protocol which connected to additional resources including local clinical pathway guidelines^a^ |
| 4. Adding further information about trigger source | 1. Added author of triggering language in evidence section of protocol and added hyperlink to source note containing triggering language^a^ |
| 5. Modifications to better reflect provider workflow | 1. Added reminder to include the genitourinary exam as part of physical exam and bolded “undressed” as reminder of important feature^a^ 2. Replaced original questions under the “Questions to Consider” heading with MORE mnemonic-based questions^e^ 3. Moved contacting Social Work earlier in the list of “Actions to Consider”/ “Available Resources”^g^ 4. Under the “Actions to Consider” heading, removed suggestion to contact local child protection team and added suggestion to consult Social Work^e^ 5. Replaced “File DCF^h^ report…” with “Ensure DCF report filed…” to reflect team effort^b,d^ 6. Removed text recommending appropriately private setting from card^g^ 7. Changed the alert to reappear at discharge to offer another opportunity for completion or addition of information^a^ 8. Added “Consider ‘unsharing’ the note ‘to prevent substantive harm to patient or another person” with tooltip with instructions on unsharing to mitigate documentation concerns^b,d^ |

- 1. Modification made to all Child Abuse Clinical Decision Support (CA-CDS) prototype versions.
  2. Modification made only in first-provider (medical or nursing provider) version of CA-CDS prototype.
  3. Modification made only in subsequent provider (medical or nursing provider) version of CA-CDS prototype.
  4. Modification was made based on feedback from third round of interviews.
  5. Modification made only in nursing provider version of CA-CDS prototype.
  6. MORE: Mechanism, Others Present, Review of Development, Examination Details
  7. Modification made only in medical provider version of CA-CDS prototype.
  8. DCF: Department of Children and Families
